# Body Mass Index and Breast Cancer Defined by Biological Receptor Status in Pre-Menopausal and Post-Menopausal Women: A Multicenter Study in China

**DOI:** 10.1371/journal.pone.0087224

**Published:** 2014-01-29

**Authors:** Jing Li, Yuan Huang, Bao-Ning Zhang, Jin-Hu Fan, Rong Huang, Pin Zhang, Shu-Lian Wang, Shan Zheng, Bin Zhang, Hong-Jian Yang, Xiao-Ming Xie, Zhong-Hua Tang, Hui Li, Jian-Jun He, Evelyn Hsieh, You-Lin Qiao, Jia-Yuan Li

**Affiliations:** 1 Department of Environmental Health and Occupational Medicine, West China School of Public Health & No.4 West China Teaching Hospital, Sichuan University, Chengdu, China; 2 Department of Epidemiology and Biostatistics, West China School of Public Health & No.4 West China Teaching Hospital, Sichuan University, Chengdu, China; 3 Department of Cancer Epidemiology, Cancer Institute & Hospital, Chinese Academy of Medical Sciences & Peking Union Medical College, Beijing, China; 4 Center of Breast Disease, Cancer Institute & Hospital, Chinese Academy of Medical Sciences & Peking Union Medical College, Beijing, China; 5 Department of Medical Oncology, Cancer Institute & Hospital, Chinese Academy of Medical Sciences & Peking Union Medical College, Beijing, China; 6 Department of Radiation Oncology, Cancer Institute & Hospital, Chinese Academy of Medical Sciences & Peking Union Medical College, Beijing, China; 7 Department of Pathology, Cancer Institute & Hospital, Chinese Academy of Medical Sciences & Peking Union Medical College, Beijing, China; 8 Department of Breast Surgery, Liaoning Cancer Hospital, Shenyang, China; 9 Department of Breast Surgery, Zhejiang Cancer Hospital, Hangzhou, China; 10 Department of Breast Oncology, Sun Yat-Sen University Cancer Center, Guangzhou, China; 11 Department of Breast-thyroid Surgery, Xiangya Second Hospital, Central South University, Changsha, China; 12 Department of Breast Surgery, the Second People’s Hospital of Sichuan Province, Chengdu, China; 13 Department of Oncosurgery, the First Affiliated Hospital of Medical College, Xi’an JiaoTong University, Xi’an, China; 14 Department of Internal Medicine, Yale School of Medicine, New Haven, Connecticut, United States of America; Wayne State University School of Medicine, United States of America

## Abstract

**Background:**

Few studies have investigated the association between body mass index (BMI) and breast cancer with consideration to estrogen/progesterone/human epidermal growth factor type 2 receptor status (ER/PR/HER2) in the breast tissue among Chinese pre- and post-menopausal women.

**Methods:**

Four thousand two hundred and eleven breast cancer patients were selected randomly from seven geographic regions of China from 1999 to 2008. Demographic data, risk factors, pathologic features, and biological receptor status of cases were collected from the medical charts. Chi-square test, fisher exact test, rank-correlation analysis, and multivariate logistic regression model were adopted to explore whether BMI differed according to biological receptor status in pre- and post-menopausal women.

**Results:**

Three thousand two hundred and eighty one eligible cases with BMI data were included. No statistically significant differences in demographic characteristics were found between the cases with BMI data and those without. In the rank-correlation analysis, the rates of PR+ and HER2+ were positively correlated with increasing BMI among post-menopausal women (*r_s BMI, PR+_* = 0.867, *P* = 0.001; *r_s BMI, HER2+_* = 0.636, *P* = 0.048), but the ER+ rates did not vary by increasing BMI. Controlling for confounding factors, multivariate logistic regression models with BMI<24 kg/m^2^ as the reference group were performed and found that BMI≥24 kg/m^2^ was only positively correlated with PR+ status among post-menopausal breast cancer cases (adjusted OR = 1.420, 95% CI: 1.116–1.808, Wald = 8.116, *P* = 0.004).

**Conclusions:**

Post-menopausal women with high BMI (≥24 kg/m^2^) have a higher proportion of PR+ breast cancer. In addition to effects mediated via the estrogen metabolism pathway, high BMI might increase the risk of breast cancer by other routes, which should be examined further in future etiological mechanism studies.

## Introduction

Breast cancer has become the most important incident cancer among Chinese women with a crude incidence rate of 41.64 per 100,000 (age-standardized rate of the world, 29.18/100,000). Between 2003 and 2007, it was the 6^th^ leading cause of death among Chinese women with a crude mortality rate of 9.63 per 100,000 (age-standardized rate of the world, 6.47/100,000) [1].

Obesity is a well-known risk factor for breast cancer development [2]. According to the China National Nutrition and Health Survey, from 1992 to 2002, the prevalence of Chinese women aged 45–59 years who were overweight and obese increased by 29.2% and 65.4% respectively [3]. In 2010, 29.7% and 12.1% of Chinese women in the general population were classified as overweight and obese respectively [4]. Based on a pooled data analysis from seven prospective cohort studies, high body mass index (BMI) was negatively correlated to breast cancer risk among pre-menopausal women while it was positively associated with breast cancer risk among post-menopausal women [2], [5]. The increased risk in post-menopausal obese women can likely be explained by the higher levels of estrogen released from endogenous estrone in adipose tissue. Multiple studies of Western populations have suggested that excess endogenous estrogen due to obesity contributes to an increased risk of both estrogen receptor (ER) and progesterone receptor (PR) positive breast cancer in post-menopausal women [6]. Yet, in Japanese women, evidence has shown that breast cancer risk with post-menopausal obesity was modified by PR status alone, not by ER status [7]. Additionally, numerous clinical studies found that breast cancers in post-menopausal obese women were prone to metastasizing and had poorer prognosis, which might be attributed to the differential expression of biological receptors in breast tissue [8], [9].

The expression of ER, PR, and human epidermal growth factor type 2 receptor (HER2) is considered to be crucial in determining prognosis and therapeutic decision making [10], [11]. Increasing evidence suggests that breast cancer subtypes defined by the expression of ER, PR, and HER2 represent distinct biological entities of breast carcinoma [12]. Based on these findings, it has been hypothesized that differing ER, PR, and HER2 receptor status may reflect different etiological mechanisms [13]. In particular, it is plausible that risk factors operating through hormonal mechanisms are differentially correlated to the breast cancer subtypes with hormone-related receptor expression [14]. As such, BMI may be more closely associated with ER and PR positivity because of the hormonally mediated mechanism of these subtypes. In contrast to findings in studies of Western women, Zheng et al. found that a lower proportion of Chinese breast cancer patients were overweight or obese, and these women were more likely to develop ER/PR negative and HER2 over-expressed breast cancer [15]. The relationship between elevated BMI and the presence of ER, PR, and/or HER2 in the breast cancer tissue of post-menopausal patients requires further evaluation.

The aim of the present study is to explore if breast cancer subtypes defined by ER, PR, and HER2 status differ according to BMI, also taking menopausal status into account, among Chinese women, thereby providing clues for further study of the etiological mechanisms of breast cancer. This study is based on the Nationwide Multicenter 10-year (1999–2008) Retrospective Clinical Epidemiological Study of Breast Cancer in China led by the Cancer Institute/Hospital, Chinese Academy of Medical Sciences (CICAMS) [16].

## Materials and Methods

### Ethics Statement

The study protocol was approved by the Institutional Review Board of the Cancer Foundation of China. The institutional review board obtained written consent from participating hospitals to access patient medical records. As it was a retrospective design, some data subjects were deceased, and it was impossible to contact with the patients or their relatives, so the Cancer Foundation of China waived the need for written informed consent from the participants.

### Study Design and Patients Collection

A nationwide multicenter clinical epidemiological study of female breast cancer over 10 years was conducted within China. All proposed data were collected retrospectively from the existing medical charts from year 1999 to 2008.

Hospital selection and case sampling procedures have been previously described elsewhere in detail [16]. Briefly, one tertiary hospital was selected in each of seven geographic regions of China: north, northeast, northwest, central, east, south, and southwest. These regions cover most of the geographic area of China and represent different levels of breast cancer burden. The leading regional public cancer hospitals were selected based on their ability to provide standardized techniques in pathological diagnosis, surgery, chemotherapy, radiotherapy, and routine follow-up care for breast cancer patients. For each year from 1999 to 2008 in each hospital, one month was randomly selected, and at least 50 pathologically diagnosed inpatient cases from that month were reviewed by trained study staff. The inpatients from each month were selected randomly to reflect the patient distribution over the whole year for each hospital. January and February were excluded from the random selection to prevent any confounding effects due to the Chinese New Year holiday. If there were fewer than 50 eligible cases in a selected month, the cases from the month immediately before and immediately after the selected month were included as well. Finally, all patients enrolled in the study had to meet 3 inclusion criteria: (1) pathological evidence of primary breast cancer; (2) inpatient admission date within the selected month in the study hospital; (3) completed or currently receiving treatment (surgery, chemotherapy, and radiotherapy) for breast cancer.

A case report form was designed to collect information from each enrolled patient’s chart regarding general information, demographic characteristics, breast cancer risk factors, breast clinical examination results, diagnostic imaging, treatment process, and pathological characteristics. Breast cancer risk factors included hormone-mediated factors (BMI, age at menopause, age at first delivery, number of live births, and breast feeding history) and non-hormone-mediated factors (family history of breast cancer), which were measured at or soon after diagnosis of breast cancer. Among them, BMI was calculated as weight (kg) divided by height squared (m^2^); family history of breast cancer was defined as breast cancer occurrence among the first-degree genetic relatives (i.e., parents, siblings, children); positive breastfeeding status indicated the patients had a more than 6-month breastfeeding experience.

### Immunohistochemistry

The status of ER, PR, and HER2 in breast cancer tissues was determined by standardized immunohistochemistry and mutual recognition at the pathology department of each selected hospital. The method and judgment criteria of immunohistochemical staining were the same across all hospitals. ER positivity (ER+) and PR positivity (PR+) were defined as at least 10% of tumor cells showing positive nuclear staining of any intensity. Samples not meeting this criteria were defined as negative (ER−/PR−) [15]. Over-expression of the HER2 gene with a score of 3+ indicated HER2 positivity (HER2+); and scores of 0, 1+ and 2+ indicated HER2 negativity (HER2−) [15], [17].

### BMI Categories

In view of the differences in BMIs among Chinese population, Western populations, and other Asian populations, we classified categories of BMI based on the Chinese adult BMI classification which was made by China Obesity Task Force and was generally accepted. [18]. Namely, BMI<24 kg/m^2^ was defined as normal, while BMI≥24 kg/m^2^ was defined as overweight in the present study.

### Statistical Analysis

The demographic characteristics of cases with and without BMI information were compared by ANOVA or chi-square test. The differences of the proportion of risk factors (age at menopause, age at first delivery, number of live births, breast feeding history, and family history of breast cancer) and pathological characteristics (pathologic type, tumor size, lymph node metastasis, and ER/PR/HER2 status) were tested by chi-squared test (or Fisher’s exact test where appropriate) between the BMI<24 group and BMI≥24 group among pre- and post-menopausal women, respectively. The relationships between BMI and ER/PR/HER2 positivity were drawn and tested by rank-correlation analysis in all, pre-, and post-menopausal cases. Taking confounding variables of other risk factors and pathological characteristics into consider, the adjusted odds ratios (ORs) and 95% confidence intervals (CI) for the relationship between BMI and ER/PR/HER2 status were estimated by the forward stepwise multiple logistic regression models (entry and exit criteria of *P* = 0.05 and *P* = 0.10) among pre- and post-menopausal women, with BMI<24 as the reference group. All statistical analyses were performed using SPSS 17.0 software with a two-tailed significance level of 0.05.

## Results

In total, 4,211 cases (48.68±10.47 years old) were selected for the original retrospective study from out of 45,200 cases seen at the 7 hospitals from 1999 to 2008. Nine hundred and thirty cases were excluded due to missing information regarding height. Ultimately, 3,281 cases (48.73±10.35 years old) with complete information were included in the analysis. (Table 1).

**Table 1:** Baseline characteristics stratified by availability of BMI results.

Demographic Characteristics	All Cases (N = 4211)		BMI Obtained Cases (N = 3281)		BMI Missing Cases (N = 930)		*F/χ* ^2^ (*P*)^a^
	N	%	N	%	N	%	
Age at diagnosis (years)							
Mean±SD	48.68±10.47		48.73±10.35		48.52±10.88		0.152 (0.859)
≤40	919	21.8	701	21.4	218	23.4	1.874 (0.759)
40–69	3126	74.2	2451	74.7	675	72.6	
≥70	166	3.9	129	3.9	37	4.0	
Age at menopause (years)							
Mean±SD	49.14±3.98		49.22±3.99		48.85±3.92		1.163 (0.313)
<50	688	16.3	522	15.9	166	17.8	6.096 (0.192)
≥50	874	20.8	706	21.5	168	18.1	
Premenopausal	2649	62.9	2053	62.6	596	64.1	
Marital status							
Single	51	1.2	42	1.3	9	1.0	11.558 (0.073)
Married	4090	97.1	3187	97.1	903	97.1	
Widowed/Divorced	52	1.2	44	1.3	8	0.9	
Unknown	18	0.4	8	0.2	10	1.1	
Education							
None	186	4.4	163	5.0	23	2.5	92.670 (<0.001)
Primary school	462	11.0	410	12.5	52	5.6	
Middle school	606	14.4	493	15.0	113	12.2	
High school	441	10.5	351	10.7	90	9.7	
University and above	396	9.4	331	10.1	65	7.0	
Unknown	2120	50.3	1533	46.7	587	63.1	

BMI = body mass index; SD = standard deviance.

a Test for heterogeneity between BMI obtained group and BMI missing group.

### Representativeness Analysis

In our study, the distribution of demographic characteristics for the 3,281 cases with BMI data and the 930 cases missing BMI data were similar. No statistical differences were observed with respect to age at diagnosis, menopausal status, and marital status between the two groups (Table 1). Though breast cases with and without BMI information had a different distribution of education, possibly affected by missing data, there were no differences observed in educational status between BMI obtained cases and the cases in total (*χ* ^2^ = 0.438, *P* = 0.995). We therefore considered the 3,281 cases with BMI information representative of the Chinese breast cancer population.

### BMI and Other Risk Factors in Pre- and Post-menopausal Women

Among all cases, age at diagnosis of breast cancer in the BMI≥24 group was older than that in the BMI<24 group. Breast cancer patients with BMI≥24 accounted for a greater percentage of post-menopausal women (44%) than pre-menopausal women (34.7%) (*χ^2^ = *28.606, *P*<0.001). A greater proportion of women with BMI<24 were nulliparous, did not have a history of breast feeding, or had a family history of breast cancer than women with BMI≥24. Compared with the BMI<24 group, women who delivered their first child at or after 30 years of age accounted for a higher proportion of the BMI≥24 group. Among the pre-menopausal cases, a higher percentage of women with BMI<24 reported late childbirth (first delivery ≥30 years old), nulliparity, and non-lactation than those with BMI≥24. However, no significant difference in rates of breast cancer family history was seen between the BMI<24 group and the BMI≥24 group. By contrast, the results from the post-menopausal cases showed no statistical differences in age at diagnosis and age at menopause between two BMI groups. However, a lower proportion of the cases with BMI<24 reported late childbirth (first delivery≥30 years old) and nulliparity than those with BMI≥24. There was no statistical difference in breast feeding history between the two BMI groups. (Table 2).

**Table 2:** BMI and other breast cancer risk factors among pre- and post-menopausal cases.

Variables	All Cases (N = 3281)		Pre-menopausal Cases (N = 2053)		Post-menopausal Cases (N = 1228)	
	BMI<24 kg/m^2^(N = 2028)	BMI≥24 kg/m^2^ (N = 1253)	BMI<24 kg/m^2^ (N = 1341)	BMI≥24 kg/m^2^ (N = 712)	BMI<24 kg/m^2^ (N = 687)	BMI≥24 kg/m^2^ (N = 541)
	N (%)	N (%)	N (%)	N (%)	N (%)	N (%)
Age at diagnosis (years)						
≤40	542(26.7)	159(12.7)	538(40.1)	159(22.3)	4(0.6)	0(0.0)
40–69	1415(69.8)	1043(83.2)	803(59.9)	553(77.7)	612(89.1)	490(90.6)
≥70	71(3.5)	51(4.1)	–	–	71(10.3)	51(9.4)
* χ^2^/Fisher* (*P*)^a^	**90.842 (<0.001)**		**65.622 (<0.001)**		3.143 (0.195)	
Age at menopause (years)						
<50	299(14.7)	223(17.8)	–	–	299(43.5)	223(41.2)
≥50	388(19.1)	318(25.4)	–	–	388(56.5)	318(58.8)
Premenopausal	1341(66.1)	712(56.8)	–	–	–	–
* χ^2^/Fisher* (*P*)^a^	**29.292 (<0.001)**		–		0.657 (0.418)	
Age at first delivery (years)						
<30	1091(53.8)	595(47.5)	704(52.5)	328(46.1)	387(56.3)	267(49.4)
≥30	72(3.6)	48(3.8)	49(3.7)	23(3.2)	23(3.3)	25(4.6)
Unknown	865(42.7)	610(48.7)	588(43.8)	361(50.7)	277(40.3)	249(46.0)
* χ^2^/Fisher* (*P*)^a^	**12.434 (0.002)**		**8.791 (0.012)**		**6.323 (0.042)**	
Number of live births						
0	47(2.3)	25(2.0)	36(2.7)	12(1.7)	11(1.6)	13(2.4)
1–2	1596(78.7)	913(72.9)	1146(85.5)	605(85.0)	450(65.5)	308(56.9)
≥3	289(14.3)	274(21.9)	85(6.3)	66(9.3)	204(29.7)	208(38.4)
Unknown	96(4.7)	41(3.3)	74(5.5)	29(4.1)	22(3.2)	12(2.2)
* χ^2^/Fisher* (*P*)^a^	**33.962 (<0.001)**		**9.367 (0.025)**		**12.568 (0.006)**	
Breast feeding history						
No	156(7.7)	61(4.9)	115(8.6)	33(4.6)	41(6.0)	28(5.2)
Yes	1248(61.5)	847(67.6)	803(59.9)	466(65.4)	445(64.8)	381(70.4)
Unknown	624(30.8)	345(27.5)	423(31.5)	213(29.9)	201(29.3)	132(24.4)
* χ^2^/Fisher* (*P*)^a^	**16.537 (<0.001)**		**12.750 (0.002)**		4.409 (0.110)	
Breast cancer family history						
No	1902(93.8)	1180(94.2)	1256(93.7)	664(93.3)	646(94.0)	516(95.4)
Yes	93(4.6)	37(3.0)	61(4.5)	25(3.5)	32(4.7)	12(2.2)
Unknown	33(1.6)	36(2.9)	24(1.8)	23(3.2)	9(1.3)	13(2.4)
* χ^2^/Fisher* (*P*)^a^	**10.941 (0.004)**		5.420 (0.067)		**7.104 (0.029)**	

BMI = body mass index;

a Test for heterogeneity in all, pre-, and post-menopausal cases respectively.

### BMI and Pathological Characteristics in Pre- and Post-menopausal Women

Among all cases, statistically significant differences were found in the distribution of pathological characteristics including invasive ductal carcinoma (IDC), HER2 status alone, and combined ER&PR&HER2 status between the BMI<24 and BMI≥24 groups. Among the pre-menopausal cases, we did not observe any statistically significant differences between the pathological characteristics of the two BMI groups. However, among the BMI≥24 subgroup of the post-menopausal cases, the proportions of PR+, as well as HER2- cases were significantly higher than among the BMI<24 group. This finding could explain the fact that after combining the status of receptors, the proportions of ER(+/−)&PR+ and ER+/PR+&HER2− cases were also statistically higher in the BMI≥24 group than that in the BMI<24 group. Moreover, there were more cases with PR+ status among the post-menopausal women than among the pre-menopausal women (*χ* ^2^ = 26.628, *P*<0.001). These findings are shown in Table 3.

**Table 3:** BMI and pathological characteristics of breast cancer among pre- and post-menopausal cases.

Variables	All Cases (N = 3281)		Pre-menopausal Cases (N = 2053)		Post-menopausal Cases (N = 1228)	
	BMI<24 kg/m^2^ (N = 2028)	BMI≥24 kg/m^2^ (N = 1253)	BMI<24 kg/m^2^ (N = 1341)	BMI≥24 kg/m^2^ (N = 712)	BMI<24 kg/m^2^ (N = 687)	BMI≥24 kg/m^2^ (N = 541)
	N (%)	N (%)	N (%)	N (%)	N (%)	N (%)
Pathologic type						
CIS	73(3.6)	41(3.3)	51(3.8)	25(3.5)	22(3.2)	16(3.0)
IDC	1694(83.5)	1015(81.0)	1114(83.1)	572(80.3)	580(84.4)	443(81.9)
Others	173(8.5)	114(9.1)	115(8.6)	66(9.3)	58(8.4)	48(8.9)
Unknown	88(4.3)	83(6.6)	61(4.5)	49(6.9)	27(3.9)	34(6.3)
* χ^2^* (*P*) a	**8.880 (0.031)**		5.510 (0.138)		3.736 (0.291)	
Tumor size						
T_1_ (≤2 cm)	589(29.0)	344(27.5)	396(29.5)	201(28.2)	193(28.1)	143(26.4)
T_2_ (2.1–5 cm)	834(41.1)	541(43.2)	511(38.1)	276(38.8)	323(47.0)	265(49.0)
T_3_/T_4_ (>5 cm)	146(7.2)	99(7.9)	102(7.6)	59(8.3)	44(6.4)	40(7.4)
Unknown	459(22.6)	269(21.5)	332(24.8)	176(24.7)	127(18.5)	93(17.2)
* χ^2^* (*P*) a	2.451 (0.484)		0.597 (0.897)		1.266 (0.737)	
LNM status						
N_0_ (0)	976(48.1)	608(48.5)	660(49.2)	347(48.7)	316(46.0)	261(48.2)
N_1_ (1–3)	548(27.0)	298(23.8)	355(26.5)	169(23.7)	193(28.1)	129(23.8)
N_2_ (4–9)	229(11.3)	152(12.1)	145(10.8)	90(12.6)	84(12.2)	62(11.5)
N_3_(≥10)	174(8.6)	126(10.1)	117(8.7)	75(10.5)	57(8.3)	51(9.4)
Unknown	101(5.0)	69(5.5)	64(4.8)	31(4.4)	37(5.4)	38(7.0)
* χ^2^* (*P*) a	5.905 (0.206)		4.547 (0.337)		4.328 (0.363)	
ER status						
ER+	1044(51.5)	666(53.2)	698(52.1)	371(52.1)	346(50.4)	295(54.5)
ER−	754(37.2)	463(37.0)	481(35.9)	261(36.7)	273(39.7)	202(37.3)
Unknown	230(11.4)	124(9.8)	162(12.1)	80(11.2)	68(9.8)	44(8.2)
* χ^2^* (*P*) a	3.735 (0.292)		0.448 (0.930)		5.997 (0.112)	
PR status						
PR+	1053(51.9)	686(54.7)	742(55.3)	392(55.1)	311(45.3)	294(54.3)
PR−	745(36.7)	443(35.4)	437(32.6)	240(33.7)	308(44.8)	203(37.5)
Unknown	230(11.4)	124(9.8)	162(12.1)	80(11.2)	68(9.8)	44(8.2)
* χ^2^* (*P*) a	4.882 (0.181)		0.551 (0.908)		**13.485 (0.004)**	
ER&PR status						
ER+&PR+	875(43.1)	562(44.9)	612(45.6)	321(45.1)	263(38.3)	241(44.5)
ER+&PR−	169(8.3)	104(8.3)	86(6.4)	50(7.0)	83(12.1)	54(10.0)
ER−&PR+	178(8.8)	124(9.9)	130(9.7)	71(10.0)	48(7.0)	53(9.8)
ER−&PR−	576(28.4)	339(27.1)	351(26.2)	190(26.7)	225(32.8)	149(27.5)
Unknown	230(11.4)	124(9.8)	162(12.1)	80(11.2)	68(9.8)	44(8.2)
* χ^2^* (*P*) a	5.382 (0.371)		0.742 (0.981)		**14.233 (0.014)**	
HER2 status						
HER2+	402(19.8)	267(21.3)	261(19.5)	152(21.3)	141(20.5)	115(21.3)
HER2−	1120(55.2)	740(59.1)	740(55.2)	410(57.6)	380(55.3)	330(61.0)
Unknown	506(25.0)	246(19.6)	340(25.3)	150(21.1)	166(24.2)	96(17.7)
* χ^2^* (*P*) a	**12.684 (0.013)**		5.359 (0.252)		**10.940 (0.027)**	
Tumor markers						
ER+/PR+&HER2−	795(39.2)	542(43.3)	537(40.0)	297(41.7)	258(37.6)	245(45.3)
ER+/PR+&HER2+	249(12.3)	157(12.5)	170(12.7)	95(13.3)	79(11.5)	62(11.5)
ER−&PR−&HER2+	150(7.4)	109(8.7)	88(6.6)	57(8.0)	62(9.0)	52(9.6)
ER−&PR−&HER2−	320(15.8)	194(15.5)	197(14.7)	110(15.4)	123(17.9)	84(15.5)
Unknown	514(25.3)	251(20.0)	349(26.0)	153(21.5)	165(24.0)	98(18.1)
* χ^2^* (*P*) a	**14.250 (0.007)**		5.944 (0.203)		**10.469 (0.033)**	

BMI = body mass index; CIS = carcinoma in situ; IDC = invasive ductal carcinoma; LNM = lymph mode metastasis;

ER = estrogen receptor; PR = progesterone receptor; HER2 = human epidermal growth factor type 2 receptor;

a Test for heterogeneity in all, pre-, and post-menopausal cases respectively.

### Rank-correlation Analysis between BMI and Receptor Status

**Figure 1:**
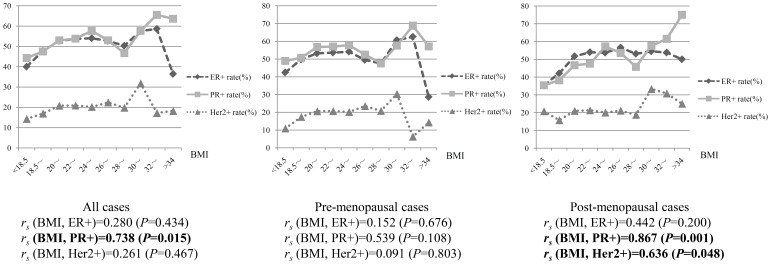
Correlations between BMI and the percentage of ER+/PR+/HER2+ in all, pre- and post-menopausal cases.

In the overall cohort, the percentage of PR+ cases increased with increasing BMI (*r_sBMI, PR+_* = 0.738, *P* = 0.015), but the percentage of ER+ and HER2+ cases did not correlate with increasing BMI. Among pre-menopausal women, no relationship was observed between the expression of ER/PR/HER2 and BMI, whereas the percentage of both PR+ and HER2+ cases were positively correlated with BMI among post-menopausal women (*r_s BMI, PR+_* = 0.867, *P* = 0.001; *r_s BMI, HER2+_* = 0.636, *P* = 0.048). The proportion of ER+ cases did not vary with increasing BMI in post-menopausal women. (Figure 1).

### Multivariate Analysis of Associations between BMI and Receptor Status

After controlling for the factors which were distributed inconsistently between the two BMI groups, multiple multivariate logistic regression models were performed to examine the associations between biological receptor status and the BMI categories, with BMI<24 as the reference group. In the post-menopausal cases, the BMI≥24 was positively correlated to PR+ status with an adjusted OR of 1.420 (95% CI: 1.116–1.808) (Wald = 8.116, *P* = 0.004). As well, the proportions of ER+&PR+ and ER−&PR+ cases were higher in the BMI≥24 group compared with the BMI<24 group among post-menopausal women. No relationship was observed between HER2 status and BMI among the entire cohort or among pre- and post-menopausal cases in the multivariate analysis. (Table 4).

**Table 4:** Multivariate logistic regression analysis of associations between BMI and breast cancer receptor status^a^.

Variables	All women^b^		Pre-menopausal women^b^		Post-menopausal women^b^	
	Adjusted *OR* (95% *CI*)	Wald (*P*)	Adjusted *OR* (95% *CI*)	Wald (*P*)	Adjusted *OR* (95% *CI*)	Wald (*P*)
ER status						
ER−	1.000	–	1.000	–	1.000	–
ER+	1.031(0.883–1.203)	0.150(0.699)	0.955(0.781–1.168)	0.199(0.655)	1.137(0.893–1.449)	1.083(0.298)
PR status						
PR−	1.000	–	1.000	–	1.000	–
PR+	1.129(0.966–1.318)	2.335(0.126)	0.965(0.787–1.184)	0.114(0.735)	**1.420(1.116–1.808)**	**8.116(0.004)**
ER&PR status						
ER−&PR−	1.000	–	1.000	–	1.000	–
ER+&PR+	1.107(0.929–1.318)	1.299(0.254)	0.957(0.762–1.202)	0.143(0.706)	**1.366(1.038–1.797)**	**4.955(0.026)**
ER+&PR−	1.008(0.759–1.339)	0.003(0.958)	1.035(0.694–1.544)	0.029(0.865)	1.153(0.666–1.495)	0.001(0.991)
ER−&PR+	1.252(0.953–1.644)	2.611(0.106)	1.046(0.739–1.481)	0.064(0.800)	**1.721(1.098–2.696)**	**5.614(0.018)**
HER2 status						
HER2−	1.000	–	1.000	–	1.000	–
HER2+	1.002(0.833–1.205)	0.001(0.986)	1.024(0.806–1.301)	0.037(0.848)	0.965(0.720–1.292)	0.058(0.810)
Tumor markers						
ER−&PR−&HER2−	1.000	–	1.000	–	1.000	–
ER+/PR+&HER2−	1.121(0.905–1.388)	1.097(0.295)	0.974(0.737–1.288)	0.035(0.852)	1.332(0.954–1.860)	2.842(0.092)
ER+/PR+&HER2+	1.023(0.801–1.384)	0.135(0.713)	0.970(0.683–1.378)	0.028(0.867)	1.132(0.730–1.756)	0.307(0.580)
ER−&PR−&HER2+	1.153(0.845–1.573)	0.805(0.370)	1.029(0.719–1.657)	0.170(0.680)	1.227(0.769–1.959)	0.735(0.391)

ER = estrogen receptor; PR = progesterone receptor; HER2 = human epidermal growth factor type 2 receptor; OR = odds ratio; CI = confidence interval;

a presents “BMI<24 kg/m^2^” as the reference group;

b age at diagnosis, age at menopause, age at first delivery, number of live births, the history of breast feeding, the family history of breast cancer, pathologic type, tumor size, LNM status, ER status, PR status, and HER2 status of breast cancer were involved in multiple forward stepwise logistic regression models with entry and exit criteria of *P* = 0.05 and *P* = 0.10; ER status, PR status, and HER2 status weren’t involved in a model twice at the same time.

## Discussion

To our knowledge, this study is the first nation-wide clinical epidemiological study of breast cancer in China to date [16]. The present study is also by far the largest study in China to explore the relationships between BMI and biological receptors among pre- and post-menopausal women, respectively. It is different from the recent published study based on the same series, which just gave the primary outline of diverse distribution of risk factors between breast cancer subtypes among whole Chinese cases [19]. Four thousand two hundred and eleven breast cancer patients were selected randomly from seven regions of China, creating a representative sample of female breast cancer cases throughout China. Due to the significant variation in BMI and ER/PR/HER2 status across the seven regions [20], the results from a multicenter study are more reliable and robust than those from a single center study. Our findings provide clues for understanding the profile of risk factors for breast cancer among Chinese females.

According to surveillance data for chronic diseases from 162 areas of 31 provinces in China, 30.6% of the Chinese population has been classified as overweight (BMI≥24 kg/m^2^) [4]. Being overweight or obese is regarded as a risk factor for breast cancer, especially for post-menopausal women. The exact mechanism for the increased risk of breast cancer in overweight or obese women is not fully understood, but it is thought that it is a function of lifetime exposure to estrogen, since the estrogen milieu is important in the initiation and progression of breast cancer lesions [21]. In post-menopausal women, the main source of estrogen is from the conversion of androgen precursor androstenedione in the peripheral adipose cell to estrogen. It is inferred that a greater amount of adipose tissue leads to greater conversion and hence greater exposure of breast cells to estrogen [22]. Conversely, in pre-menopausal women, obesity may protect against breast cancer by causing more frequent anovulatory menstrual cycles, which may reduce overall exposure to estrogen in these women [5], [21]. In our study, risk factors operating through hormonal mechanisms were distributed differently between the two BMI groups among pre- and post-menopausal women. Post-menopausal breast cancer cases with BMI≥24 reported more late childbirth and nulliparity. In contrast to pre-menopausal women, the pathogenesis of post-menopausal breast cancer may be more related to exposure to estrogen and progesterone. The interactions of BMI and reproductive factors require further epidemiologic evaluation in the future.

Multiple studies seeking to evaluate the molecular basis behind these relationships have found that the association between obesity and breast cancer risk varies by menopausal status, and may be attributable to the status of ER and PR in breast tissues [6], [13], [23]. ER and PR, the nuclear hormone receptors, exist in normal breast epithelial cells to regulate breast development by hormones during puberty and pregnancy and are usually present in low quantities [24], [25]. Hormones stimulate the rate of breast epithelial cell proliferation, perhaps via combination with the over-expression of ER and PR. Cell proliferation is essential for carcinogenesis because cell division increases the risk of errors during DNA replication, which if not corrected, may lead to the development of cancer [26]. In general, PR status is strongly correlated with the expression of ER, because PR is considered an ER− regulated gene and its presence indicates a functioning ER pathway [27]. Hence, it has been hypothesized that ER+&PR+ responsive tumors might be more strongly associated with the risk factors that are likely mediated by endogenous hormones, while ER−&PR− tumors might be inversely associated with these risk factors [28]. Previous Western studies had found that high BMI was most clearly associated with ER+ and/or PR+ tumors [6]. In the Shanghai Breast Cancer Study, excessive BMI was associated with an increased risk of the ER+&PR+ subtype but not the ER−&PR− subtype among postmenopausal women [14]. In the present study, we found a positive relationship between being overweight (BMI≥24 kg/m^2^) and PR+ status in breast tissue among the post-menopausal cases by both univariate analysis and multivariate analysis. However, elevated BMI was not related to ER+ breast cancer among post-menopausal women nor ER+/PR+ breast cancer among pre-menopausal women. These results are consistent with findings from Japan [7] as well as a recent meta-analysis from the United States [13]. Moreover, the same relationship between high BMI and PR+ status was seen in a large population based study of endometrial carcinoma, which is similarly modified by estrogen [29]. The distinct results between the ER and PR status suggest that an alternate signaling pathway may down-regulate the expression of PR gene [27]. In addition to effects mediated by the estrogen metabolism pathway, high BMI might also increase the risk of developing breast cancer via other routes.

With regards to HER2 status, biological studies have suggested that the status of HER2 could antagonize the expression of hormone receptor [27], [30]. The loss of PR in some tumors was due to excessive growth factor receptor signaling (over-expression of HER2) [27]. Consistent with these findings, our univariate analysis suggested that the proportions of HER2+ cases were statistically different between the two BMI groups in the overall cohort and in the post-menopausal women. More cases with HER2- tumor were seen in the BMI≥24 group than that in the BMI<24 group, however multivariate analysis did not yield the same results.

As the different trends of ER+/PR+/HER2+ breast cancers among heavily obese patients were apparent in Figure 1, BMI was classified into three categories based on the Chinese anthropometric parameters: BMI<24, 28>BMI≥24, and BMI≥28, which represents normal, overweight, and obesity, respectively. However, any significant difference of risk factors and pathological characteristics between the overweight group and the obese group was not found by using chi-square test. Otherwise, in multivariate regression analysis, there was no significant association between the biological receptor status and the BMI≥28 category but the same tendency as that in the 28>BMI≥24 group. The fewer cases in BMI≥28 group (303/3279) might cause the insufficient power to find the significance in multivariate analysis. The lack of a significant difference between the overweight group and the obese group may be an interesting negative result and further study should focus on those heavily obese patients with breast cancer.

Obesity has been shown to be a poor prognostic factor in post-menopausal patients already diagnosed with breast cancer [8]. Being overweight at or soon after diagnosis of breast cancer was related to lower rates of overall survival and disease-free survival among Chinese women [31]. In the present study, we did not find any significant differences in tumor size and the number of lymph mode metastases between the BMI<24 group and the BMI≥24 group. However, the distribution of PR status was statistically different between the two BMI groups. Post-menopausal obese women had a higher proportion of PR+ tumor. PR exists as two isoforms, PR-alpha and PR-beta. The excessive production of PR-beta is associated with increased breast cancer risk, whereas overabundance of PR-alpha is related to resistance to tamoxifen. Clinical studies have found that the ER−&PR+ subtype of breast cancer is not highly sensitive to tamoxifen. Possible interaction between BMI and PR status that was responsible for poorer prognosis of post-menopausal obese breast cancer patients might exist. Some experts have postulated that the PR status or the ratio of PR-alpha to PR-beta can more accurately predict the therapeutic benefit of tamoxifen and prognosis of breast cancer [32]. Competitive antagonists of progesterone might be considered as a novel way to cure the PR+ breast carcinoma in post-menopausal obese women [33].

This study has potential limitations. First, due to the original study design, there was no comparison group of normal healthy women with whom to compare the risk factors for developing breast cancer. Case-case analysis was not possible to explore the risk factors for cancer but we were able to describe the difference between breast tumor subtypes. Second, data quality was dependent on the thoroughness of the clinician’s documentation and some cases were missing risk factor information, especially regarding history of smoking, alcohol use, and oral contraceptive use, which could have interactions with BMI. Third, poor accessibility of Fluorescence in situ hybridization (FISH) analysis for HER2 in some of the study hospitals resulted in missing values and may account for the inability to detect significant differences for HER2 expression between different BMI groups in multivariate analysis. Fourth, the Waist-Hip Ratio (WHR) is considered a superior index for evaluating central obesity, which is common among post-menopausal women. However no information was collected regarding waist circumference and hip circumference for the breast cancer patients in the study. Nevertheless, BMI remains a useful tool and recent studies from Japan and America have begun to focus on evaluating the association of BMI gain from a younger age and the risk of breast cancer onset [34], [35].

## Conclusion

In summary, the results of this study showed that among post-menopausal Chinese women with elevated BMI (BMI≥24 kg/m^2^), there was an increased proportion of PR+ breast cancer. However, the proportion of ER+ cases did not vary with increasing BMI in post-menopausal women. In addition to effects mediated by the estrogen metabolism pathway, high BMI might also increase the risk of developing breast cancer via other routes, which should be further evaluated in future etiological mechanism studies.
